# Role of glia in delirium: proposed mechanisms and translational implications

**DOI:** 10.1038/s41380-024-02801-4

**Published:** 2024-10-27

**Authors:** Áine Bríd Heffernan, Moritz Steinruecke, Georgia Dempsey, Siddharthan Chandran, Bhuvaneish T. Selvaraj, Zoeb Jiwaji, Maria Stavrou

**Affiliations:** 1 UK Dementia Research Institute at The University of Edinburgh, The University of Edinburgh, Edinburgh, UK; 2 Centre for Clinical Brain Sciences, The University of Edinburgh, Edinburgh, UK; 3 University of Cambridge School of Clinical Medicine, Cambridge, UK; 4 School of Medicine, University of St Andrews, St Andrews, UK; 5 Centre for Neural Circuits and Behaviour, University of Oxford, Oxford, UK; 6 Euan MacDonald Centre for Motor Neuron Disease Research, The University of Edinburgh, Edinburgh, UK; 7 Anne Rowling Regenerative Neurology Clinic, The University of Edinburgh, Edinburgh, UK

**Keywords:** Neuroscience, Psychiatric disorders

## Abstract

Delirium is a common acute onset neurological syndrome characterised by transient fluctuations in cognition. It affects over 20% of medical inpatients and 50% of those critically ill. Delirium is associated with morbidity and mortality, causes distress to patients and carers, and has significant socioeconomic costs in ageing populations. Despite its clinical significance, the pathophysiology of delirium is understudied, and many underlying cellular mechanisms remain unknown. There are currently no effective pharmacological treatments which directly target underlying disease processes. Although many studies focus on neuronal dysfunction in delirium, glial cells, primarily astrocytes, microglia, and oligodendrocytes, and their associated systems, are increasingly implicated in delirium pathophysiology. In this review, we discuss current evidence which implicates glial cells in delirium, including biomarker studies, post-mortem tissue analyses and pre-clinical models. In particular, we focus on how astrocyte pathology, including aberrant brain energy metabolism and glymphatic dysfunction, reactive microglia, blood-brain barrier impairment, and white matter changes may contribute to the pathogenesis of delirium. We also outline limitations in this body of work and the unique challenges faced in identifying causative mechanisms in delirium. Finally, we discuss how established neuroimaging and single-cell techniques may provide further mechanistic insight at pre-clinical and clinical levels.

## Introduction

Delirium is characterised by acute onset disturbances and fluctuations in attention, awareness and cognition [1, 2]. It affects over 20% of all medical inpatients and up to 74% of patients in the intensive care unit (ICU) [3–5]. Key risk factors for developing delirium include patient characteristics, such as elderly age, dementia, frailty and multimorbidity, and health events, including trauma, surgery, new medications and drug withdrawal are common triggers [6]. Therefore, in the context of ageing populations in many high-income countries, delirium poses a significant challenge to healthcare systems.

Delirium develops over 24–72 h and can be classified as hypoactive, hyperactive or mixed depending on patients’ clinical presentation. The syndrome usually resolves within days but can last weeks to months. Some patients do not return to their cognitive baseline following episodes. In addition, delirium is independently associated with accelerated cognitive decline, increased hospital costs, and mortality [3, 5, 7, 8]. The healthcare consequences of delirium were recently highlighted during the SARS-CoV-2 pandemic, when one in three hospital inpatients developed delirium, which was associated with three-fold higher mortality [9]. Despite this clinical and social significance, delirium remains relatively understudied [10].

The underlying pathophysiology of the syndrome is poorly understood and there is a lack of effective pharmacological interventions [11, 12]. This is compounded by its heterogenous aetiology and clinical presentation. Therefore, there is an urgent need for an improved understanding of the neurobiology of delirium, with the aim of developing diagnostic and therapeutic strategies.

Many studies adopt a neuron-centric approach to delirium and aim to understand changes in brain network and neurotransmitter activity or to identify biomarkers of neuronal damage, such as neuron-specific enolase and phosphorylated neurofilament heavy subunit [13–16]. However, these approaches do not consider the complex neuro-glial interactions which may be disrupted in delirium, and notably, accumulating experimental data suggests a role for glial cells, including astrocytes, microglia and oligodendrocytes, in delirium pathogenesis [17, 18]. In this review, we evaluate current evidence which supports a role for glial cells and systems in the pathogenesis of delirium and outline directions for future research.

## Studying the pathophysiology of delirium

As a fluctuating cognitive syndrome with a heterogenous aetiology, the underlying pathophysiology of delirium can be challenging to study [19]. Post-mortem studies only capture the end-stage of the disorder and methods typically used to assess delirium in humans, such as cognitive testing, are difficult to adapt to animal models. Neuroimaging approaches often lack the resolution required to form conclusions on the cellular and molecular pathology underlying delirium and biofluidic analyses often focus on single metabolites and proteins. In addition, the pathological effects of episodes of delirium can be challenging to isolate in patients with a high number of comorbidities. However, epidemiological evidence consistently shows that there is a significant role for patient susceptibility, such as ageing or a diagnosis of a neurodegenerative disorder, in delirium [6, 8].

**Fig. 1:**
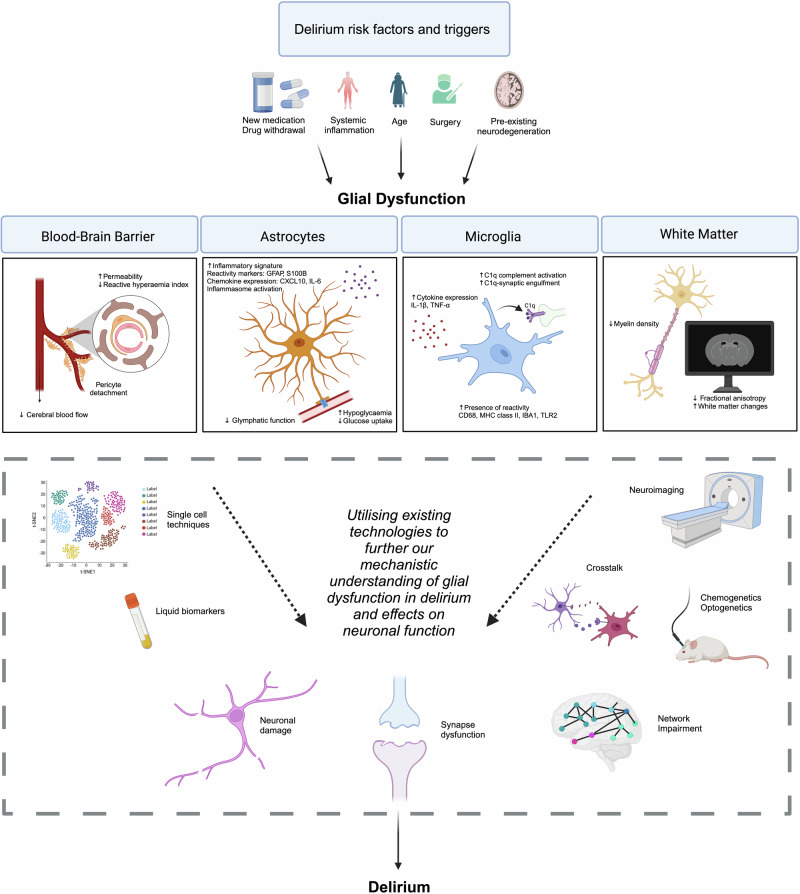
Schematic outline of glial dysfunction in delirium and technologies which can be used to further our understanding of their roles in its pathogenesis. Glial dysfunction has been described in delirium, but the underlying pathophysiological mechanisms are not fully understood. Key risk factors for and triggers of delirium include older age, pre-existing neurodegeneration, systemic inflammation, surgery, new medications and drug withdrawal. Reactive astrocytes with increased inflammasome activity, altered metabolism and impaired glymphatic activity have been reported in patients and models with delirium. Similarly, microglia display elevated cytokine production and C1q-tagged signalling. Impaired blood-brain barrier function and white matter changes have also been noted. Exploiting existing technologies including single-cell transcriptomics, neuroimaging, liquid biomarkers and optogenetics would provide deeper mechanistic insight into the roles of dysregulated glia in delirium. The effects of these mechanisms on neuronal function should also be interrogated. GFAP: glial fibrillary acidic protein; CXCL10: C-X-C motif chemokine ligand 10; IL-6/1β: interleukin-6/1β; TNFα: tumour necrosis factor α; C1q: complement component 1q; CD68: cluster of differentiation 68; TLR2: toll-like receptor 2.

Therefore, animal models frequently use aged rodents or those that model neurodegenerative disorders and subject them to “secondary insults”, such as central or systemic inflammation (e.g. lipopolysaccharide [LPS] injection) or post-operative conditions (e.g. laparotomy). Naturally, these models vary in their precise cellular and molecular pathology since they are based on different aetiologies but nevertheless often implicate common pathways. Rodent models have replicated delirium-like behaviour and display neuroimmune signatures which are mediated by astrocytes and microglia [20, 21]. This is in line with evidence of increased astrocytic and microglial reactivity with age and in neurodegeneration and suggests that delirium may exacerbate this phenotype [22–24]. Together, animal models suggest that cellular priming, defined as enhanced cellular reactivity on the background of pre-existing pathology, plays an important part in the pathogenesis of delirium. In the following sections, we discuss pre-clinical and clinical studies which provide evidence to suggest that glial cell types and systems contribute to the pathophysiology of delirium (Fig. 1).

## Role of astrocytes in delirium

Astrocytes have diverse physiological functions which include regulating neuronal energy supply, modulating synaptic activity, contributing to neurogenesis, supporting the blood-brain barrier (BBB) and maintaining ion and neurotransmitter homeostasis. Many of these processes are impaired in delirium, but whether they are direct contributors remains unclear. Challenges also arise when studying astrocytic reactivity in this disorder since it can have both protective and pathological phenotypes [25]. In this section, we discuss the evidence for structural astrocyte pathology, aberrant brain energy metabolism and abnormalities in the glymphatic system in delirium.

### Astrocytic inflammatory signatures in delirium

Astrogliosis is frequently observed in models of delirium and has been noted in patients with delirium. A post-mortem study identified raised astrocytic glial fibrillary acid protein (GFAP) immunoreactivity in patients with a history of delirium compared to age-matched controls, primarily in the dentate gyrus [26]. In addition, raised levels of S100B, a protein primarily expressed by astrocytes, is frequently associated with an increased risk of developing delirium following hospital admission [27].

Pre-clinical studies have provided further insight into this inflammatory signature. In an aged mouse model of post-operative delirium, astrocytes were noted to have a reactive morphology with an atrophied cell soma and shorter, deramified processes [28]. When the amyloid precursor protein/presenilin 1 mouse model of Alzheimer’s disease (AD) was subjected to a secondary inflammatory challenge with interleukin-1β (IL-1β), primed astrocytes developed exaggerated chemokine responses [21]. Similar results were observed in a model of tauopathy injected with LPS, which displayed astrogliosis, acute sickness behaviour and accelerated tau pathology [24]. In a model of post-operative delirium, reactive astrocytes showed aberrant NOD-like receptor protein 3 inflammasome activation and γ-aminobutyric acid (GABA) synthesis [29].

Several molecules with immunomodulatory properties have shown therapeutic effects in rodent models via astrocytic mechanisms. Dexmedetomidine, an a_2_-adrenoceptor agonist, displayed neuroprotective effects following LPS challenges by reducing astrocytic pyroptosis and inflammasome activation [30]. Minocycline, an anti-inflammatory drug, reduced inflammatory signatures in astrocytes and long-term cognitive impairment in a model of post-operative delirium [31]. Targeting β-arrestin1, which interacts with dynamin-related protein 1 to regulate mitochondrial fusion/fission and has reduced expression in reactive astrocytes, has also shown promising pre-clinical results [32]. These approaches warrant further pre-clinical and translational investigation.

### Aberrant brain energy metabolism in delirium

Astrocytes play key roles in brain energy metabolism, including via the astrocyte-neuron lactate shuttle. Patients with clinical risk factors for developing delirium, particularly a diagnosis of AD, often have pre-existing deficits in brain energy metabolism. The expression of GLUT1, a glucose transporter, is reduced in AD and patients display impaired glucose utilisation and insulin sensitivity [33–35]. Animal studies show that mice with pre-existing neurodegeneration are vulnerable to hypoglycaemia, which is associated with delirium in both clinical and experimental settings [36–38]. A 2-^18^F-fluoro-2-deoxyglucose positron emission tomography (PET) study identified reduced brain-wide glucose metabolism in patients with delirium [39]. In an animal model of septic encephalopathy, glucose uptake was preferentially reduced in the neocortex, which was associated with decreased cerebral blood flow (CBF) and alpha activity on electroencephalography [40]. Although definitive cellular resolution is not yet possible with this technique, rodent work suggests that this mechanism is likely driven by glutamate uptake by astrocytes [41]. Additionally, in one rodent model of post-operative delirium, when glial metabolism was inhibited with fluorocitrate for three days following surgery, GFAP expression was reduced and there were improvements in cognitive function [42].

A recent study showed that patients who developed delirium following a hip fracture had increased concentrations of ketone bodies in their cerebrospinal fluid (CSF) compared to those who did not develop delirium but that there was no difference in CSF or serum glucose concentrations [43]. Astrocytes are known to regulate the concentration of ketones in the brain via fatty acid oxidation [44]. Several studies have also identified raised CSF lactate levels in patients who developed delirium following a significant fracture, but this relationship may be mediated by patients’ age and comorbidities [36, 43]. Patients with delirium lasting more than five days have been found to have higher CSF lactate levels than outpatients diagnosed with dementia [45]. In addition, metabolic conditions, such as hepatic encephalopathy, in which hyperammonaemia induces osmotic dysregulation in astrocytes via the glutamate-glutamine cycle, mimic some clinical features of delirium [46–51]. In a rodent model of hepatic encephalopathy, astrocytes were found to be enlarged and displayed increased GFAP, tumour necrosis factor α (TNFα) and aquaporin-4 (AQP4) expression and reduced Kir 4.1 immunoreactivity [52]. Together, these findings are suggestive of altered brain glucose utilisation in patients with delirium, which may be mediated by astrocytes.

There is limited evidence on whether modulating these metabolic signatures in astrocytes would be clinically feasible or effective. For example, valproic acid (VPA), a medication currently used for bipolar disorder and epilepsy, is believed to act via GABA signalling and may also modulate excitatory/inhibitory imbalances via astrocytes [53, 54]. In addition, VPA has been shown to ameliorate pro-inflammatory signatures in astrocytes, including reducing GFAP levels [55, 56]. There are several reports of VPA being used in antipsychotic-resistant cases of delirium, but these are largely uncontrolled studies and robust evidence on this question is lacking [57]. Further studies on the potential role of VPA in the management of delirium may provide insight into the mechanisms of astrocytic dysfunction in the syndrome.

### Abnormalities in the glymphatic system

The glymphatic system is comprised of a network of perivascular channels formed by astrocytes, with AQP4 channels located on astrocyte endfeet being an important component of the system [58]. In vivo models of neurodegenerative diseases, such as AD, have identified impairments in the glymphatic system [59]. It has been suggested that glymphatic impairment also occurs in the post-operative period, which could contribute to cognitive disturbances, but this remains largely unexplored [60].

Anaesthetic agents are known to affect glymphatic function and astrocytic reactivity, which may contribute to post-operative delirium [61–63]. Sevoflurane, an inhaled anaesthetic, disrupts calcium currents in astrocytes, which may be important in regulating glymphatic flow [64]. Sevoflurane is also associated with raised cerebral glucose and lactate levels, which correlate with post-operative delirium scores in children [65]. Other anaesthetic agents, such as dexmedetomidine and propofol, appear to promote glymphatic activity [61, 66, 67]. Dexmedetomidine also reduces the expression of monocyte chemoattractant protein-1, a pro-inflammatory cytokine, in LPS-induced astrocytes, ameliorates excessive GABA receptor expression, and is associated with a lower incidence of post-operative delirium than other anaesthetic agents [68–70]. The glymphatic system represents a possible therapeutic target for delirium, especially post-operatively, but further studies are needed. In particular, the use of aged *aqp4* knockout mice subjected to surgery or systemic inflammation could provide further insight. However, assessing glymphatic function in pre-clinical models remains challenging as methods are highly invasive and animals need to be anaesthetised for imaging. In addition, certain aspects of the glymphatic system are not yet well-established in humans and can be difficult to assess in clinical studies [71].

## Role of microglia in delirium

Microglia are the resident immune cells of the brain and remove apoptotic neurons, release inflammatory mediators, respond to local tissue factors and fine tune neural circuitry during post-natal development [72–74]. Dysregulation of pro-inflammatory and pro-reparative signals from microglia, for example during neurodegeneration, result in neuronal dysfunction, cell death and synapse loss [75]. In this section, we discuss evidence for microglial reactivity and dysregulated  homeostasis  in delirium.

### Microglial reactivity and dysregulated microglial  homeostasis  in delirium

Post-mortem studies have identified raised expression of the myeloid lineage markers human leukocyte antigen-DR and cluster of differentiation 68 in the brain tissue of patients with a history of delirium, independent of a co-existing infection or diagnosis of dementia [26]. Brain tissue of patients with a history of sepsis or sepsis-associated encephalopathy displayed a similar phenotype, including complement component 1q (C1q) activation in hippocampal regions [76, 77]. Proteomic studies of CSF samples from patients with delirium have also identified several inflammatory markers which are associated with microglia. For example, fractalkine levels are raised in the CSF of patients with infectious delirium and AD when compared to healthy controls [78]. Fractalkine is a chemokine ligand which binds to CX3C chemokine receptor 1 (CX3CR1) on microglia and regulates microglial reactivity [79]. In contrast, both CD200 receptor 1 (CD200R1) and caspase 8 (CASP8) are downregulated in patients with infectious delirium and AD. CD200 is expressed by neurons and binds to CD200 receptors expressed on microglia to maintain a microglial resting state by regulating cytokine production [80, 81]. CASP8 is a ligand involved in cell apoptosis and inhibits pro-inflammatory microglial activity [82]. Soluble triggering receptor expressed on myeloid cells 2 (TREM2), a receptor found on microglia, is also found at higher levels in the CSF of patients with delirium in the absence of pre-existing dementia, which highlights the importance of stratifying delirium patients by their dementia status [83].

Pre-clinical studies have provided some additional mechanistic detail. Microglia isolated from aged mice subjected to LPS challenges display increased expression of IL-1β, MHC class II molecules, toll-like receptor 2 (TLR2) and IL-10 [84]. Cytokine challenges with TNFα or IL-1β and polyinosinic:polycytidylic acid injections, which mimic systemic viral infection, also result in microglial priming in mouse models of neurodegeneration [85, 86]. Microglial reactivity, measured by increased IBA1 expression, has been observed in the hippocampi of mice subjected to LPS and was associated with an imbalance between pro- and anti-inflammatory cytokines [87]. The authors suggested that this effect may be mediated by TLR4, a LPS receptor, as blocking TLR4 signalling with viral inhibitory peptide for TLR4 (VIPER) attenuated LPS-induced neuroinflammation. Additionally, in rodents, depleting microglia by inhibiting the colony-stimulating factor 1 receptor (CSF1R) protects against post-operative cognitive decline via an anti-inflammatory mechanism [88, 89]. CSF1R inhibition has also been shown to reduce C1q complement activation and protect neurons from microglial engulfment, which is associated with cognitive improvements in mice [77].

Several studies have used minocycline, an established anti-inflammatory drug which targets NF-κB, to reduce microglial reactivity in models of delirium [90, 91]. One study demonstrated that minocycline reduces post-operative microglial reactivity and decreases the expression of IL-1β and TNFα in rat hippocampi [92]. A similar effect has been shown for astrocytes [31]. Another group showed a reduction in cytokine expression and microglial reactivity in the hippocampus following administration of minocycline and an IL-1 receptor antagonist [93]. This signature was also observed when minocycline was administered following an LPS insult, which resulted in reduced cytokine and microglial TLR2 surface expression [94]. Similarly, artemisinin, an anti-malarial drug with anti-inflammatory properties, has been shown to reduce LPS-induced cognitive impairment, likely by activating AMPKα1 signalling in microglia and suppressing pro-inflammatory cytokine production [95].

However, many of these anti-inflammatory drugs are generic immunomodulators which act on many cell types and these studies do not show whether decreased microglial reactivity was a direct result of the drug or a subsequent reduction in LPS- or surgery-mediated damage. Recent clinical trials of prophylactic minocycline administration in at-risk patients undergoing major surgery have showed contrasting effects on post-operative cognitive impairment [96, 97]. Several other molecules with anti-inflammatory properties have displayed improvements in microglial reactivity, inflammatory signatures and cognitive impairment in rodent models of delirium and warrant further translational studies [98–102].

## Blood-brain barrier impairment in delirium

The BBB is a multicellular structure comprised of astrocytes, pericytes, neurons, microglia, endothelial cells and the basement membrane. The BBB has several important functions, including delivering nutrients, removing waste and regulating immune functions, all of which are impaired in delirium [103]. The precise contribution of BBB impairment to delirium remains unclear and assessment of BBB integrity can provide widely varying results depending on the methodology used [104].

BBB permeability, measured by the CSF:plasma albumin ratio, correlates with post-operative delirium incidence, severity, and delayed recovery [105, 106]. Notably, these studies showed that short-term increases in BBB permeability from before surgery to 24 hours after surgery are more significant in patients who develop post-operative delirium compared to those who do not, even when accounting for other clinical factors. Patients with elevated levels of plasma markers of endothelial cell activation and BBB injury, such as E-selectin and plasminogen activator inhibitor-1, are at increased risk of experiencing prolonged episodes of delirium [107]. Critically ill patients with a lower reactive hyperaemia index, an indicator of poorer systemic endothelial cell function, also experience prolonged periods of acute brain dysfunction [108].

BBB impairment is well-characterised in ageing and can drive excessive transforming growth factor-β signalling in astrocytes, which leads to neuronal dysfunction in humans and rodent models [109]. Astrocytes mediate signalling between neurons and the vasculature and increase CBF in response to neuronal activity. A significant reduction in whole-brain and regional CBF has been demonstrated in patients experiencing delirium, which resolved when symptoms improved [110]. This reduction in CBF has also been identified in a rat model of septic encephalopathy and was associated with reduced cerebral glucose uptake [40]. In addition, transcranial ultrasound studies have described abnormal cerebral autoregulation in patients experiencing delirium [111]. These mechanisms are likely associated with previously discussed findings of abnormal glucose utilisation in the brain during delirium and may represent a link between astrocytic reactivity, metabolic dysfunction and BBB impairment.

Pre-clinical studies have also identified structural BBB pathology, particularly changes in tight junction proteins and the basal lamina, in models of delirium [28]. In an LPS-induced mouse model of septic encephalopathy, pericytes detached from the basal lamina, which was associated with increased cerebrovascular permeability [112]. In vitro, cultured mouse cerebrovascular endothelial cells challenged with blood plasma from septic mice displayed a dissociation of occludin from the cytoskeleton and increased permeability [113]. An AD-related model of post-operative delirium demonstrated changes in AQP4 and GFAP expression in astrocytes following surgery and increased extravasation of fibrinogen and dextran across the BBB [114]. Proximity labelling techniques, such as TurboID, would allow for protein interactions, for example with astrocytic endfeet or endothelial cells, at the BBB to be studied, providing greater mechanistic detail.

Further research is also needed to determine whether therapies targeted toward the BBB can reduce delirium risk and improve prognosis. Explorative studies indicate that statins, which can modify the endothelium and reduce inflammation, may be protective. Evidence from a prospective cohort study suggests that statin use amongst critically ill patients is associated with a lower incidence of delirium [115, 116]. Controlled trials and long-term cognitive outcome studies would be needed to support this. Preventing the recruitment of inflammatory monocytes across the BBB could be an attractive therapeutic target for delirium and a pre-clinical model showed that this was associated with reduced neuroinflammation and cognitive impairment [117]. While administration of neuroprotectin D1, a docosahexaenoic acid-derived lipid mediator, has also been shown to ameliorate BBB pathology and was associated with improvements glial function and memory [28]. Additionally, microfluidic and multicellular organoid models of the BBB have the potential to provide further insight into possible prophylactic and treatment options by enabling high-throughput screening of candidate molecules [118]. Interestingly, therapies targeting the BBB could also be non-pharmacological and a small study showed that early physical therapy may improve endothelial function and reduce delirium duration in patients admitted to an ICU [119].

## White matter dysfunction in delirium

Oligodendrocytes are glial cells which produce and maintain myelin in the central nervous system and provide metabolic support to surrounding neurons. Overall, there is mixed evidence on the role of white matter abnormalities, including hyperintensities, fractional anisotropy and altered diffusivity, in acute cognitive impairment. In particular, the extent to which white matter changes predispose patients to developing delirium, are involved in the syndrome itself, or are simply incidental findings is unclear.

White matter hyperintensities have been identified in up to 75% of patients with delirium but are also common in healthy aged individuals [120]. In the Oxford Vascular Study, patients with a history of stroke or transient ischaemic attack who also had white matter changes on magnetic resonance imaging (MRI) were more likely to develop delirium up to five years following their cerebrovascular incident [121]. This study also showed that white matter changes were associated with future delirium risk, independent of pre-admission cognitive status. In addition, in a cohort of patients undergoing cardiac surgery, those who displayed reduced pre-operative fractional anisotropy, a readout of white matter impairment, were at increased risk of developing post-operative delirium [122]. In a recent pre-clinical study, aged mice which were cognitively vulnerable to a systemic inflammatory challenge displayed increased microglial reactivity in white matter regions and the degree of their cognitive dysfunction correlated with the extent of myelin pathology around the hippocampal formation [123]. Conversely, a study of patients admitted within 48 hours of the onset of stroke symptoms identified comparable white matter changes in most brain regions between patients who developed delirium and those who did not [124]. Other studies have described no relationship between white matter lesions and post-operative delirium [125–127].

Some studies have considered the potential contribution of white matter changes to clinical outcomes and long-term sequalae. Specifically, white matter abnormalities identified on MRI have been shown to correlate with clinical outcomes in patients with sepsis and delirium [128]. A diffusion tensor imaging study described reduced fractional anisotropy in ICU patients with prolonged episodes of delirium [129]. These imaging features were present three months following discharge and were associated with worse cognitive outcomes up to one year later. A recent study of patients admitted with SARS-CoV-2 infection and associated neurological symptoms, including delirium, identified volume shifts indicative of vasogenic oedema affecting white matter tracts and correlated the magnitude of these findings with cognitive impairment [130].

Further neuroimaging studies are required to detect structural white matter changes which may contribute to episodes of delirium. Using data from prospective cohorts, for example the UK Biobank, would allow for thorough investigation of delirium risk factors and long-term outcomes.

## Future directions

Both pre-clinical and clinical studies have identified dysregulated glial cells and systems in delirium, but in many cases, the cellular mechanisms underlying this pathology are unclear. In addition, the crosstalk between neurons and glia in delirium and how this may be disrupted is largely unexplored (Table 1).

**Table 1:** Summary of dysregulated glial phenotypes and their hypothesised effect on neuronal function.

Dysregulated glial phenotype	Hypothesised effect on neuronal function
Increased astrocytic and microglial reactivity associated with enhanced proinflammatory cytokine production [21, 31, 84, 87]	Pro-inflammatory cytokines can induce neuronal apoptosis, alter synaptic function, and facilitate vascular dysfunction [138–141]
Reduced glucose uptake and cerebral blood flow [36–40]	Reduced energy supply to neurons, which can lead to excitotoxicity-mediated cell death [142]
Glymphatic impairment [60]	Accumulation of metabolic toxins and waste products can impair neuronal function [58, 59]
White matter dysfunction associated with white matter hyperintensities and reduced fractional anisotropy [120, 128, 129]	Reduced speed and quality of signals within networks [143–145]
Blood-brain barrier dysfunction and hyperpermeability [28, 105]	Entry of toxins, pathogens and peripheral immune cells into the brain via a leaky blood-brain barrier can damage neuronal networks and dysfunctional efflux via this barrier also impairs the removal of toxins and waste products [103, 117]

Techniques such as fluorescence-activated cell sorting to isolate astrocytes, microglia or oligodendrocytes from animal models of delirium, followed by transcriptomic or proteomic analyses would help elucidate cell type-specific pathway dysregulation. Single-cell isolation, for example, via laser capture microscopy of post-mortem tissue or enzymatic dissociation of rodent brains, could be used to explore the role of regional heterogeneity and identify cell clusters which drive disease pathogenesis. Targeting a subset of heterogenous cell types may also improve therapeutic efficacy, since generic anti-inflammatory treatments have generally not been successful [131].

Aberrant metabolic processes in astrocytes in delirium could be explored further using existing metabolic modulators. For example, ceftriaxone, which promotes glutamate transport, a function which is likely disrupted in astrocytes during delirium, could be used in animal models to assess effects on cognition [41]. Astrocytes and microglia play important roles in modulating the neural circuitry and future work could investigate this using calcium imaging and opto- and chemogenetic tools in delirium models [132]. Therefore, applying technologies currently used to investigate glial dysfunction in other neurological disorders could help elucidate the roles of these cell types in delirium.

While there is some clinical evidence which suggests a role for white matter impairment in delirium, this has not been explored extensively in pre-clinical models. Changes in white matter could be assessed at different time points in delirium pathogenesis using MRI, along with cognitive testing as a correlative investigation. Compounds associated with protection or repair of white matter could be tested in these models to determine if this therapeutic strategy is associated with improved cognitive function.

The impaired crosstalk between glial cells in delirium also warrants further investigation. For example, it has been demonstrated that reactive microglia, by secreting IL-1α, TNFα and C1q, induce a unique phenotype in astrocytes in neurodegenerative disorders such as Alzheimer’s and Parkinson’s disease [133]. Targeting this aberrant signalling to prevent neurotoxic crosstalk between microglia and astrocytes has been shown to be protective in models of Parkinson’s disease and could offer a new therapeutic approach for delirium [134].

Several neuroimaging methods have the potential to improve our clinical understanding of the roles of glial cells in delirium aetiology and progression. Two PET tracers, 11C-BU99008, which primarily binds to I2-imidazoline-2 on the outer mitochondrial membrane in astrocytes, and 11C-DED, which primarily targets monoamine oxidase B in astrocytes, are currently in use and could provide further insight into the metabolic changes in astrocytes during episodes of delirium [135]. The utility of astrocytic and microglial plasma biomarkers, including chitinase-3-like protein 1, translocator protein (18 kDa), CX3CL1 and CSF1, which are currently being trialled for other disorders should also be explored in the context of delirium [19, 136, 137]. The development of tracers which can discriminate between different glial phenotypes would allow for investigation of these dynamic states throughout the course of the syndrome and in response to treatment. Finally, additional studies using dynamic contrast-enhanced MRI and PET, for example targeting P-glycoprotein-mediated efflux at the BBB, are needed to elucidate the temporal contribution of BBB impairment to delirium.

## Conclusion

Delirium remains an understudied condition, especially given its high prevalence of approximately one in five medical inpatients. The pathophysiology of delirium is poorly understood and no effective pharmacological treatments which target its pathogenesis have been identified. Emerging evidence has described dysfunctional glial cells and systems in delirium. This includes the loss of astrocytic functions in brain energy metabolism and the glymphatic system, aberrant microglial reactivity, impairment of the blood-brain barrier, and white matter dysfunction. However, mechanistic detail in these areas is still lacking. We propose that exploiting existing technologies, such as high-resolution neuroimaging and single-cell approaches, would provide insight into the spatiotemporal involvement of glia in delirium and identify dysregulated pathways which could be targeted. Establishing glial biomarkers is also important for improving our understanding of the roles of these cells in delirium risk, prognosis and management. A better understanding of the role of glia in delirium across multiple pre-clinical and clinical models will help advance the development of effective therapeutic strategies.
